# The roles of tissue-resident macrophages in sepsis-associated organ dysfunction

**DOI:** 10.1016/j.heliyon.2023.e21391

**Published:** 2023-10-30

**Authors:** Yulei Gao, Xin Tian, Xiang Zhang, Grace Divine Milebe Nkoua, Fang Chen, Yancun Liu, Yanfen Chai

**Affiliations:** a Department of Emergency Medicine, Tianjin Medical University General Hospital, Tianjin, 300052, P. R. China; b Department of Emergency Medicine, China-Congo Friendship Hospital, Brazzaville, 999059, P. R. Congo; c Department of Medical Research, Beijing Qiansong Technology Development Company, Beijing, 100193, P. R. China; d Department of Medical Research, Sen Sho Ka Gi Company, Inba-gun, Chiba, 285-0905, Japan; e Department of Emergency Medicine, Rizhao People's Hospital of Shandong Province, Rizhao, 276825, P. R. China

**Keywords:** Sepsis, Septic shock, Sepsis-associated organ dysfunction, Tissue-resident macrophages

## Abstract

Sepsis, a syndrome caused by a dysregulated host response to infection and characterized by life-threatening organ dysfunction, particularly septic shock and sepsis-associated organ dysfunction (SAOD), is a medical emergency associated with high morbidity, high mortality, and long-term sequelae. Tissue-resident macrophages (TRMs) are a subpopulation of macrophages derived primarily from yolk sac progenitors and fetal liver during embryogenesis, located primarily in non-lymphoid tissues in adulthood, capable of local self-renewal independent of hematopoiesis, and developmentally and functionally restricted to the non-lymphoid organs in which they reside. TRMs are the first line of defense against life-threatening conditions such as sepsis, tumor growth, traumatic-associated organ injury, and surgical-associated injury. In the context of sepsis, TRMs can be considered as angels or demons involved in organ injury. Our proposal is that sepsis, septic shock, and SAOD can be attenuated by modulating TRMs in different organs. This review summarizes the pathophysiological mechanisms of TRMs in different organs or tissues involved in the development and progression of sepsis.

## Highlights

• TRMs are the first line of defense against life-threatening conditions, including sepsis.
• The function of the TRMs has two sides - the angelic and the demonic - in sepsis.
• For protection against sepsis and SAOD, TRMs should be the focus of research.

## 1 Introduction

Sepsis remains a leading cause of health loss worldwide and the costliest condition for healthcare systems [[1], [2], [3], [4]]. In a study of the global burden of disease from 1990 to 2017, an estimated 48.9 million (38.9～62.9) cases of sepsis were recorded worldwide in 2017, and 11.0 million (10.1～12.0) sepsis-associated deaths were reported in the same year, accounting for 19.7 % (18.2～21.4) of global deaths [1]. The incidence and mortality rates of sepsis vary widely among regions, with the highest burden of disease in sub-Saharan Africa, Oceania, South Asia, East Asia, and Southeast Asia [1,5]. In septic patients with septic shock and sepsis-associated organ dysfunction (SAOD), protocols to improve sepsis recognition, antimicrobial stewardship, and early source control have been used to improve prognosis. This is expected to improve survival in all sepsis cases studied, but the effect is not uniform [2,3,[6], [7], [8]]. A retrospective analysis of septic patients based on discharge records showed that multiorgan failure occurred in 21.9 % of cases, with the most common organ failure being renal (56.8 %), followed by cardiovascular (24.2 %) [2]. Among septic patients who received antibiotics within 3 h, the risk of death for patients with septic shock had increased by 35 % for every 1 h of delay in antibiotic administration [3]. Septic shock, SAOD, sepsis recurrence, and the presence of comorbidities are independently associated with increased mortality, particularly in the intensive care unit (ICU) [[2], [3], [4],^8^].

Tissue-resident macrophages (TRMs) are a subpopulation of macrophages in tissues characterized by organ heterogeneity, homogeneity of origin (mainly from the yolk sac- and the fetal liver-derived macrophages), and local self-renewal independent of hematopoiesis during adulthood (Table 1) [[9], [10], [11], [12], [13], [14]]. TRMs are essential for immune defense, angiogenesis, tissue repair and development, and the maintenance of homeostasis mainly through phagocytosis, antigen presentation, and production of cytokines, inflammatory and anti-inflammatory signals, growth factors, and protein hydrolases [[14], [15], [16], [17], [18]].

**Table 1:** Characterization of the major tissue-resident macrophage subpopulations.

Organ	Origin	Subset	Premier Phenotype	Primary function	Primary function in sepsis
Brain	Yolk sac	Microglia	F4/80^+^ CD11b^+^ Iba1^+^ CD45^low^	Promoting normal neuronal development and function Remodeling of synapses Monitoring of the immune system	Initiating primary inflammatory responses
Heart	Yolk sac	Embryonic macrophage	CCR2－ MHC-II^low^ TIMD4＋ LYVE1－ CCR2－ MHC-II^high^ TIMD4^+^ LYVE1^+^ CX3CR1＋ CCR2－ Ly6C－ MHC-II＋ CD14＋ CD45＋ CD64＋ CCR2－ HLADR^high^	Maintaining intracardiac homeostasis	Inhibiting excessive inflammatory responses Removing dysfunctional mitochondria Preventing metabolic disorders Maintaining cardiac electrical activities
Lung	Fetal liver	Alveolar macrophage	CD68^+^ F4/80^+^ CD11C^+^ Siglec-F^+^ MARCO^+^	Surveillance of Immunity Homeostasis of surfactant	Initiating primary inflammatory responses Involving in immunosuppression
Peritoneal cavity	Yolk sac	Large peritoneal macrophage	F4/80^high^ CD11b^+^ MHC-II^low^	Immune surveillance Removal of death cells	Reducing plasma and peritoneal bacterial load
	Fetal liver	Small peritoneal macrophage	CD11b^+^ F4/80^low^, MHC-II^high^	Immune surveillance	Exacerbating the excessive inflammatory response
Vasculature	Fetal liver Yolk sac	Perivascular macrophage	CD45^+^ F4/80^+^ CD38^+^ LYVE1^+^ FOLR2^+^ CD206^high^	Regulating vascular permeability Maintaining vascular integrity Promoting vascular development	Inducing endotoxin tolerance Reducing pathological behavior
Spleen	Yolk sac	White pulp macrophage	MerTK^+^ Tim-4^+^	Regulating the function of B cells	
	Yolk sac	Red pulp macrophage	F4/80^+^ VCAM^+^	Phagocytosis of old and damaged red blood cells Degradation of heme Recycling of iron	
	Fetal liver	Marginal zone macrophage	MACRO^+^ SIGN-R1^+^	Immune surveillance	Clearing bloodborne pathogens
	Fetal liver	Marginal zone metallophilic macrophage	CD68^+^CD169^+^	Immune surveillance	Clearing bloodborne pathogens
Liver	Yolk sac-derived erythromyeloid progenitor	Kupffer cell	F4/80^high^ CD11b^low^ CD169^+^ CD68^+^ CD80^low^	Removing blood-borne particles and commensals Supporting metabolism Tissue repair Immune surveillance	Inducing irreversible hepatocyte damage Clearing bloodborne pathogens

**Fig. 1:**
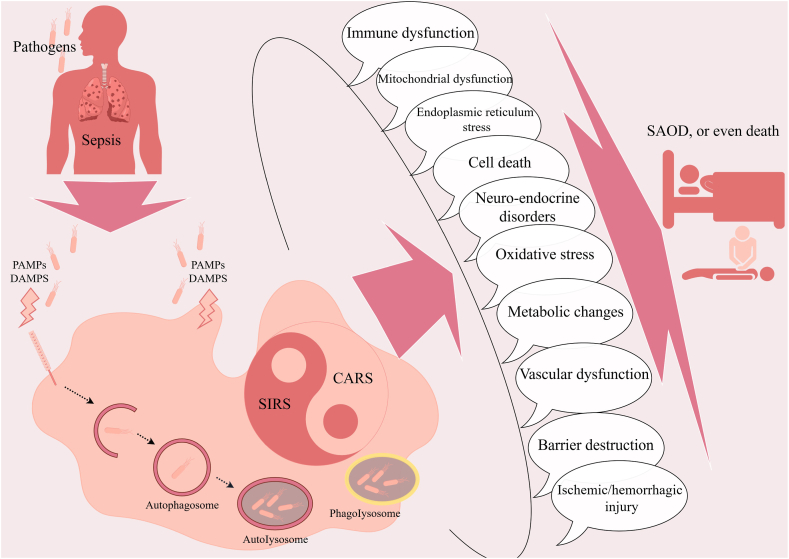
The major pathophysiologic mechanisms of sepsis-associated organ dysfunction. CARS: compensatory anti-inflammatory response syndrome; DAMPs: damage-associated molecular patterns; PAMPs: pathogen-associated molecular patterns; SAOD: sepsis-associated organ dysfunction; SIRS: systemic inflammatory response syndrome.

Sepsis can be divided into two stages, early and late, based on immunologic characteristics [[19], [20], [21]]. Simultaneously, complex signaling pathways such as nuclear factor (NF)-κB, extracellular signal-regulated kinase/mitogen-activated protein kinase (ERK/MAPK), signal transducer and activator of transcriptions, signal transduction through the mothers against decapentaplegic, suppressor of cytokine signaling, stimulator of interferon genes protein (STING) are involved [19,[21], [22], [23], [24]]. The first three days of sepsis are defined as the early stage of sepsis, characterized by a systemic inflammatory response syndrome (SIRS) [25,26]. Most patients survive the SIRS-induced “cytokine storm” in the early stage and then enter the later stage which is dominated by compensatory anti-inflammatory response syndrome (CARS) [19,21]. The mechanisms of organ dysfunction caused by sepsis include not only SIRS and CARS, but also dysfunction of the immune-inflammatory response, mitochondrial dysfunction, endoplasmic reticulum stress, oxidative stress, and cell death that occur locally in the organs (Fig. 1) [[27], [28], [29], [30], [31], [32]]. Although bone marrow-derived tissue-infiltrating macrophages play a primary and crucial role in the pathophysiological mechanisms of sepsis, it is clear with the application of technologies such as single-cell RNA sequencing (scRNA-seq) and *in vivo* cell tracking that some vital TRMs play a protective role in SAOD by clearing damaged mitochondria, expressing pro-inflammatory cytokines [*e.g.*, interleukin (IL)-1] receptor antagonists, inhibiting pro-inflammatory cytokine secretion (*e.g.*, IL-6), forming immune cell scaffolds, maintaining cellular homeostasis, promoting tissue repair, and other mechanisms, and even showing potential therapeutic value [[32], [33], [34], [35], [36], [37]]. However, there is also a subset of TRMs that are activated by components of pathogens such as lipopolysaccharide (LPS), and exacerbate SAOD through mechanisms such as oxidative stress, secretion of pro-inflammatory cytokines, and vascular leakage [[38], [39], [40], [41], [42]]. Thus, with two sides - angelic and demonic - the role of TRMs in the pathophysiological mechanisms of sepsis is complex and unclear.

This review provides a comprehensive overview of different types of TRMs and common organ dysfunction caused by sepsis. We briefly summarize the origin of different TRMs, their phenotypes, and their functions in different organs. In particular, we focus on the pathophysiological mechanisms of TRMs involved in the progression of sepsis and SAOD. We will discuss how sepsis disrupts the crosstalk between TRMs and tissue cells in different organs to maintain organ homeostasis and how TRMs protect the human body against SAOD, as well as their potential therapeutic value. Our search strategy was essential as follows: “TS= (resident macrophage*) AND TS= (sepsis × OR septic*)".

### 1.1 Sepsis-associated encephalopathy and microglia

#### 1.1.1 Sepsis-associated encephalopathy

Sepsis-associated encephalopathy (SAE) is a common diffuse central nervous system (CNS) dysfunction that often occurs in the setting of sepsis, septic shock, or SIRS [[43], [44], [45], [46], [47]]. In the case of sepsis, the dysfunction of the immune-inflammatory response (neuroinflammation) served as the root of the major pathological mechanisms of ischemic/hemorrhagic injury, blood-brain barrier breakdown, cerebrovascular dysfunction, and metabolic alterations. In the brain, the dysfunction of the immune-inflammatory response can be divided into two categories: primary responses caused by the resident immune cells and secondary responses induced by the peripheral immune cells [43,45].

#### 1.1.2 Microglia

Microglia, a population of CNS-resident macrophages, are long-lived yolk sac-derived cells within the CNS parenchyma that remain in a stable state throughout adulthood and self-renew without the contribution of bone marrow cells [48]. Microglia, as the core of immune regulation in monitoring the normal function of the brain and maintaining CNS homeostasis, are involved in many critical CNS functions, ranging from gala-, vasculo-, and neurogenesis to the growth and integrity of synapses and myelin sheath through their process dynamics, the release of soluble mediators and phagocytosis ability [48,49]. This means that microglia are crucial for understanding the pathophysiological mechanisms of most CNS diseases.

#### 1.1.3 Activation of microglia worsens the development and progression of sepsis-associated encephalopathy

In the context of infection, microglia undergo significant morphological transitions in response to pathogen-associated molecular patterns (PAMPs), such as LPS, and initiate primary inflammatory responses [45,48]. In the septic environment, due to the intense secretion of pro-inflammatory factors and chemokines, the blood-brain barrier is severely damaged, and the peripheral immune cells such as neutrophils and macrophages can be recruited and infiltrated into the CNS, especially the hippocampi, which further enhance the response and activation of microglia and even oxidative stress and endoplasmic reticulum stress-associated inflammation and apoptosis via the expression of inflammatory mediators such as tumor necrosis factor (TNF)-α, IL-1β, IL-18, and IL-6 exacerbating neuroinflammation and brain injury [19,43,45,47,50]. The most common clinical manifestation of SAE is cognitive impairment, as well as coordination and sensorimotor dysfunction [[43], [44], [45]]. The brain's ability to accurately control a person's movements depends on the synchronization of cerebellar Purkinje cells [51]. Sepsis activates microglia and promotes the production of pro-inflammatory factors (TNF-α), which increases the intrinsic excitability and synaptic transmission of cerebellar Purkinje cells and ultimately impairs motor learning and behavior [46].

### 1.2 Septic cardiomyopathy and cardiac-resident macrophages

#### 1.2.1 Septic cardiomyopathy

Septic cardiomyopathy (SCM), a life-threatening complication, is usually considered a cardiohomeostasis disorder caused by sepsis and septic shock as long as physicians can rule out other types of chronic and acute cardiovascular diseases [52,53]. According to previous studies, the presence of SCM indicates a worse prognosis and significantly increases the patient mortality rate to 70～90 % [53]. In contrast, the mortality rate associated with SCM was 35.6 % in a recent study [2]. This may be related to the development of ultrasound technology, especially the use of point-of-care ultrasound by emergency physicians, and the fact that complex pathophysiological mechanisms are gradually being elucidated [21,[52], [53], [54], [55]]. The pathological and physiological mechanisms of SCM are complex [52]. However, the key mechanism of cardiac homeostasis disruption in the early stage of sepsis is excessive inflammation, such as macrophage infiltration and pro-inflammatory cytokines, which depends on the crosstalk among cardiomyocytes, cardiac fibroblasts, endothelial cells, and immune cells, followed by mitochondrial injury, metabolic disorders, and cell death, such as apoptosis, pyroptosis, and autophagy [29,55].

#### 1.2.2 Cardiac-resident macrophages

The cardiac-resident macrophages (CRMs), derived from yolk sac progenitors, are continuously self-renewing with a turnover period of nearly 5 weeks, which contributes to the maintenance of cardiac homeostasis, such as immune surveillance, angiogenesis, antifibrosis, electrical conduction, adaptive myocardial remodeling, clearance and degradation of apoptotic cardiomyocytes [34,[56], [57], [58], [59]]. In the rodent heart, according to the expression levels of *C*–C chemokine receptor type 2 (CCR2), T-cell immunoglobulin, mucin domain containing 4 (TIMD4), major histocompatibility complex-II (MHC-II), and lymphatic vessel endothelial hyaluronan receptor 1 (LYVE1), the subsets of CRMs, derived from the yolk sac, are CCR2－MHC-II^low^TIMD4＋LYVE1－ and CCR2－MHC-II^high^TIMD4^+^LYVE1^+^ [14]. In the cardiac ontogeny process of neonatal mice, *C*-X3-C motif chemokine receptor (CX3CR) 1＋CCR2－Ly6C－MHC-II－CRMs directly promote the proliferation of cardiomyocytes through the jagged-1-Notch1 axis, thus ameliorating cardiac injury. CX3CR 1＋CCR2－Ly6C－MHC-II－CRMs can be converted into CX3CR1＋CCR2－Ly6C－MHC-II＋CRMs by changes in the cellular redox state, which is the main population in the adult mouse heart [9]. It shows that the CCR2 negative is a characteristic marker of CRMs. In the human heart, there are also similar characteristics of CRMs, such as CD14＋CD45＋CD64＋CCR2－HLADR ^high^CRMs [11].

#### 1.2.3 Cardiac-resident macrophages prevent and ameliorate the disruption of cardiac homeostasis in septic cardiomyopathy

There is a growing body of direct evidence supporting the theory that CRMs could maintain cardiac homeostasis and protect patients from SCM by inhibiting excessive inflammatory responses, removing dysfunctional mitochondria shed by cardiomyocytes, preventing metabolic disorders, and maintaining cardiac electrical activities [34,60,61].

Like the dysfunction of other organs, SCM is caused by a dysregulation of the host response to the infection, involving many pathways of the infectious inflammatory response, especially the excessive local cardiac and systemic inflammatory response in the early stage of sepsis, driven by PAMPs and damage-associated molecular patterns (DAMPs) [21,52]. Similar to SCM, the imbalance of oxygen supply and demand in the human body causes acute cardiovascular disease, particularly acute myocardial infarction (AMI), which results in a violent excessive inflammatory response characterized by leukocyte infiltration of the heart [52,53,62,63]. The subsets of CRMs mediate myocardial tissue inflammation and repair after AMI, while the excessive inflammatory response can also damage the function of CRMs, even reducing the differentiation, development, self-renewal, and total number of CRMs [9,58,64]. Therefore, much work has been done to find new and promising therapies targeting the excessive inflammatory response after AMI, the cardioprotective properties of CRMs, and even chronic inflammatory responses such as cardiac fibrosis and heart failure [62,65,66]. In the cecal ligation and puncture (CLP)-induced sepsis model, the number of CX3CR1＋CCR2－CD64＋CRMs begins to decrease from the first day of sepsis, which is associated with TNF-α and IL-6 signaling and leukocyte infiltration. It returns to normal levels by day 28, and 93.8 % of this is self-renewal from locally derived cells [63]. Similarly, this phenomenon occurs simultaneously in triggering receptor expressed on myeloid cells (TREM) 2^high^ CD163＋ resistin-like alpha (RETNLA)＋, MHC-II^low^, and MHC-II^high^ CRMs [34,60].

The heart is a highly energy-consuming organ [67,68]. Most of the adenosine triphosphate (～95 %) used by the heart is synthesized by the mitochondrion, mainly by oxidative phosphorylation (mainly fatty acid β-oxidation), and is known as the “powerhouse” or “energy factory” of cardiomyocytes [68]. A growing body of research indicates that mitochondrial dysfunction is a critical feature of sepsis and septic shock and contributes to the development of SAOD [21,27,67]. Therefore, prevention and amelioration of mitochondrial dysfunction, removal of dysfunctional mitochondria from an organ, and other mitochondrial-based therapeutic strategies are being developed to address the sequelae of sepsis, septic shock, and SAOD, as evidenced by some clinical trials [24,27,34,45,53,67,[69], [70], [71], [72]]. The mitochondrial dysfunction, mainly including ultrastructural and structural protein damage, oxidative damage, defects of the electron transport chain and oxidative phosphorylation complexes, dysregulation of calcium homeostasis, overproduction of mitochondrial reactive oxygen species (ROS), mitochondrial death (especially autophagy and apoptosis), and so on, is life-threatening damage that leads to the cardiac homeostasis disorder in the case of sepsis [29,52,73,74]. Therefore, maintaining cardiac mitochondrial homeostasis is very important for the recovery from SCM. A network of CRMs supports cardiac function. Myocardial cells shed dysfunctional mitochondria into the exoplasmic vesicle, which must be eliminated by specialized CRMs, which collectively support proper cardiac function, enhance myocardial repair, and maintain cardiac mitochondrial homeostasis [64,68,75]. The TREM2^high^ CD163＋RETNLA＋CRMs subpopulation has an active ability to scavenge cardiomyocyte-ejected mitochondria, which is critical for supporting cardiac function and promoting cardiac homeostasis under septic stress, according to a recent study that combined scRNA-seq with fate-mapping techniques to analyze dynamic changes in CRMs in a CLP-induced septic heart model [34].

### 1.3 Sepsis-associated vascular injury and perivascular macrophages

#### 1.3.1 Sepsis-associated vascular injury

Sepsis-associated vascular injury (SAVI) is a life-threatening pathophysiological feature of septic shock and acute organ injury, including vascular hyporesponsiveness, vascular relaxation, hypotension, leakage, microcirculatory abnormalities, disseminated intravascular coagulation, and even circulatory failure [6,27]. Vasculature is a general term for the tubes that flow through the blood and form the circulatory system with the heart, including arteries, veins, and capillaries [76]. The function of the vasculature is mainly to transport various nutrients through vascular mechano-transduction, to meet the body's needs for activities, and to remove metabolites from the body through the metabolism of lungs, kidneys, liver, *etc* [77]. Thus, the improvement of SAVI may lead to the reversal of the deterioration of sepsis and its associated complications, such as the protection of vascular integrity and tension, the regulation of vascular reactivity and permeability, and the modulation of vascular homeostasis [30,40,78]. The maintenance of vascular homeostasis, integrity, tension, reactivity, and permeability relies on the mechanotransduction of vascular smooth muscle cells (VSMCs) and vascular endothelial cells (VECs), including their sensing of mechanical stimuli and the transduction of mechanical signals, particularly in VECs [77]. In the case of sepsis, the dysfunction of VECs and VSMCs, which is caused by the hyper-inflammatory response (both systemic and organ local), vascular inflammation, oxidative stress, mitochondrial dysfunction, endoplasmic reticulum stress, immune thrombosis, and accumulation of metabolites such as lactic acid, is the root cause of SAVI, and even the interactions between VECs, which are special types of immune cells, and immune cells (especially macrophages) or, VSMCs are also involved [27,30,35,40,63,78].

#### 1.3.2 Perivascular macrophages

Perivascular Macrophages (PVMs), the highly organically heterogeneous and organ-specific subpopulation of macrophages, are located within the vessel wall, on the surface of the vessel lumen, or close to the vessel [79,80]. In addition to the organ-specific features of TRMs, such as peritoneal resident macrophages (PRMs), Kupffer cells (KCs), microglia, alveolar macrophages (AMs), and CRMs, PVMs have other characteristic markers that contribute to the maintenance of vascular homeostasis and self-renewal, such as olfactory receptor-2, and cellular musculoaponeurotic fibrosarcoma [81,82].

#### 1.3.3 Perivascular macrophages could maintain vascular homeostasis in sepsis

In addition to phagocytosis and antibody presentation, PVMs play a role in maintaining vascular homeostasis by directly interacting with VECs and VSMCs to regulate permeability, maintain vascular integrity, and promote vascular development [40,80,81,83,84]. The blood labyrinth barrier is recognized as a vascular endothelial barrier consisting of the basement membrane, branched PVMs, pericytes, and endothelial cells that adhere to each other through tight junctions; thus, the integrity of the blood labyrinth barrier is critical for hearing function [40,80]. In one study, researchers injected LPS through the tympanic membrane of hearing-impaired adult Balb/c mice for 48 h, and the mice exhibited vascular leakage and loss of integrity in the cochlear vascular striae associated with reduced contact between PVMs and capillaries and a reduced number of tight junction contact points between PVMs, pericytes, and endothelium as a direct result of LPS [39]. This finding is consistent with the previously defined pattern of F4/80＋GSTα4＋perivascular-resident macrophage-like melanocytes (PVM/Ms), *i.e.* expressing characteristics of both monocytes and macrophages [38]. In sepsis, circulating endotoxins such as LPS-induced sickness behaviors in the CNS, such as fever, decreased activity, and reduced food and water intake, while intracranial PAMs contribute to the rapid production of pro-inflammatory cytokines, especially IL-1β, in subfornical organ of the brain and induce endotoxin tolerance that attenuates sickness behaviors [44,45,47,50,85]. Intracerebroventricular administration of the IL-1β-saporin conjugate dose does not exacerbate sickness behavior or cause more severe SAE, except for a reduction in brain PAMs [44,86].

### 1.4 Acute lung injury/acute respiratory distress syndrome and alveolar macrophages

#### 1.4.1 Acute lung injury/acute respiratory distress syndrome

According to the Berlin Criteria, acute lung injury (ALI) or acute respiratory distress syndrome (ARDS), caused by non-pulmonary sepsis, severe pneumonia, aspiration of gastric contents, trauma, severe pancreatitis, burns, aspiration injury, near-drowning, multiple transfusions, shock, and other unusual conditions, is characterized as acute hypoxic respiratory failure with bilateral chest radiographic infiltrates and cannot be fully explained by heart failure or fluid overload [[87], [88], [89]]. There is a direct causal relationship between ARDS and mortality in trauma [89].

#### 1.4.2 Alveolar macrophages

AMs, the lung resident macrophages, originate and develop from the fetal liver during embryonic hematopoiesis mainly through granulocyte-macrophage colony-stimulating factor, peroxisome proliferator-activated receptor-γ, and transforming growth factor-β-mediated signaling pathways and self-renewal, which facilitates the maintenance of the abundance of AM and serves as the first line of defense of the airways [90]. During the progression of ALI or ARDS, AMs are critical to both the initiation and the resolution of inflammatory responses via the crosstalk between AMs and alveolar epithelial cells [91,92].

#### 1.4.3 The role of alveolar macrophages in acute lung injury/acute respiratory distress syndrome is characterized by a double-edged sword

The severity of organ injury in sepsis is determined by changes in macrophage phenotype, specifically the pro-inflammatory phenotype (M1 subtype) and the anti-inflammatory phenotype (M2-like subtype) [93]. In the early stage of sepsis-induced ALI or ARDS, AMs are activated, polarized, and transformed into the M1-like subtype, which directly induces lung injury via the excessive release of toxic species such as pro-inflammatory cytokines, chemokines, and oxidative stress, and indirectly induces lung injury via the recruitment of neutrophils and Th17 cells to the lung [[91], [92], [93], [94], [95], [96]]. Although macrophages, as well as AMs, are characterized by a switch to the M2-like subtype in the late stage of sepsis, the host is in an immunosuppressive state [21]. After the resolution of severe sepsis or septic shock, AMs exhibited poor phagocytosis and self-renewal for several weeks [97]. This suggests that AMs are also involved in the immunosuppressive phase and even lead to long-term lung immunoparalysis. Most patients with ARDS can survive in the acute stage, but many of them succumb to death due to progressive pulmonary fibrosis [98]. However, based on the observations from Bao and colleagues, suppressing the shift of AMs to the M1-like subtype-AMs phenotype while promoting the shift of AMs to the M2-like subtype-AMs phenotype could prevent and treat sepsis-associated pulmonary fibrosis [99].

Clinical observations show that primary sites of infection are the most important determinants of sepsis severity, particularly in the lung, abdomen, and bloodstream [2,89,100]. For example, in non-pulmonary (CLP-induced) and pulmonary (*Escherichia coli*-induced) septic mouse models, researchers performed the double hit of intratracheal challenge with *Pseudomonas aeruginosa* and found that non-pulmonary septic mice, characterized by defective lung bacterial clearance and increased mortality rate, are highly susceptible to double-hit, while pulmonary septic mice are the opposite. The root cause of this experiment result is that non-pulmonary and pulmonary sepsis modulated the amount of AMs and some important immune functions differently via Toll-like receptor (TLR) 2-dependent crosstalk between Regulatory T cells and AMs [101].

ALI or ARDS is associated with impaired AM efferocytosis: the reduction of AM efferocytosis in sepsis-induced ARDS patients correlates with increased alveolar inflammation, which may contribute to serious clinical outcomes, including mortality [102]. Activation of the inflammasome, particularly the nucleotide-binding oligomerization domain and leucine-rich repeat pyrin domain-containing protein (NLRP)-3, is central to the process of uncontrolled inflammation and the pathogenesis of sepsis and sepsis-associated ALI or ARDS [95,103]. The AMs-depleted mice, challenged with CLP and followed by pulmonary transplantation of p120-catenin-deficient macrophages, greatly enhanced the accumulation of IL-1β and IL-18 in bronchoalveolar lavage fluid, which is associated with overactivation of the NLRP3 inflammasome [103]. In addition, during the progression of CLP-induced ALI or ARDS, the infiltration of neutrophils into the lung tissue causes the release of neutrophil extracellular traps and IL-1β, which upregulate the level of NLRP3, NLRP3 inflammasome assembly, and caspase-1 activation in AMs, leading to the AMs pyroptosis and sustain lung injury [95]. Conversely, mitophagy-promoting miR-138–5p promoter demethylation subsequently inhibits NLRP3 inflammasome, AMs pyroptosis, and sepsis-induced ALI or ARDS in a CLP mouse model [72]. These results suggest that ALI or ARDS caused by non-pulmonary sepsis is associated with the overactivation of NLRP3 inflammasome in AMs, and silencing NLRP3 inflammasome can alleviate AMs pyroptosis and lung injury.

### 1.5 Abdominal bacterial infection, sepsis, and peritoneal-resident macrophages

#### 1.5.1 Peritoneal-resident macrophages

PRMs, which typically include large peritoneal macrophages (LPMs) and small peritoneal macrophages (SPMs), are long-term resident macrophages that develop well only in the peritoneal cavity [10,12,23]. LPMs could differentiate during embryonic life and maintain themselves by self-renewal during adult life [23]. LPMs are derived from the fetal yolk sac [12,23,104]. *Myb* is a critical transcription factor for the self-renewal of hematopoietic stem cells (HSCs). LPMs have been shown to be independent of the development of the HSCs and the transcription factor *Myb* [12,104]. Under pathological conditions, PRMs are the first line of defense against life-threatening abdominal sepsis in the peritoneal cavity. However, excessive activation of PRMs can lead to a hyper-inflammatory state that facilitates tissue damage and organ dysfunction [22,23,36]. PRMs play a critical role in repairing peritoneal damage and controlling microbial and parasitic infections [23]. In particular, Bhlhe40, the GATA6-driven transcription of PRMs, is regulated by extracellular signals associated with pathological conditions, including abdominal sepsis [23,37,105]. However, we need to further elaborate on the content of sepsis in this section.

#### 1.5.2 Large peritoneal macrophages contribute to the prevention and amelioration of sepsis

Both animal experiments and clinical studies show that the ERK/MAPK signaling pathway plays a critical role in the pathogenesis of sepsis and septic shock [19,21,22]. Sprouty-related EVH1-domain-containing proteins (Spreds) belong to a family of proteins that inhibit the ERK signaling pathway, whereas Spred2-deficient proteins increase leukocyte infiltration by upregulating the ERK/MAPK signaling pathway and exacerbate LPS-induced pneumonia [94]. Surprising and confusing! In the early stage of the abdominal polymicrobial sepsis model, the survival rate of the Spred2^−/−^ mice is improved, accompanied by increased peritoneal infiltration of leukocytes (neutrophils and macrophages) and local proinflammatory cytokines/chemokine production (IL-6, CXCL1, and CCL2), and reduced plasma and peritoneal bacterial loads. These effects are attributed to the enhanced ERK/MAPK signaling pathway and complement receptor 1/2 (CR1/2) expression of PRMs following Spred2 gene knockout [22]. During the self-renewal of LPMs, monocytes enter the peritoneum and differentiate into SPMs [10,106]. In the peritoneum of the CLP-induced septic mouse model, LPMs (CD11b^+^, F4/80^hi^, MHC-II^low^) are rapidly increased in the peritoneum from 18 to 66 h after CLP. Surprisingly, SPMs (CD11b^+^, F4/80^lo^, MHC-II^hi^) are not increased until 14 days after CLP. Changes in monocyte numbers by *CCR2* deficiency or adoptive transfer of monocytes did not significantly affect animal survival. Depletion of LPMs significantly increases the mortality of the CLP model [37,106]. These results suggest that LPMs are essential for sepsis survival in the early stage of sepsis [19,23,106,107]. At the same time, some shreds of evidence, such as macrophage migration inhibitory factor (MIF)-2, also showed that the increased number and functions of SPMs exacerbated abdominal sepsis [108].

In the experiments, *Myb*^−^LPMs reduced the severity of sepsis and SAOD such as liver and lung, improved the survival rate of septic rats by 30 %, and reduced the bacterial load by enhancing bacterial phagocytosis [104]. Stem cell antigen (Sca-1) is a characteristic marker of the HSCs [109]. Sca-1^+^Gr-1^+^myeloid cells exacerbated death in the *S. aureus* infection model. Meanwhile, by using the LPS-induced sepsis model, they further confirmed that the number of Sca-1 was only increased in macrophages. Sca-1^+^ macrophages developed from PRMs. LPS-induced Sca-1^+^PRMs formation was partially interrupted by an *anti*–IFN–γ antibody, indicating that IFN-γ was necessary for this process. Compared with the sca-1^−^macrophages, the LPS-stimulated products such as IL-6, TNF-α, and CCL2 were lower in Sca-1^+^PRMs. Depletion of Sca-1^+^PRMs significantly increased survival rate and reduced acute organ injury [110].

#### 1.5.3 Large peritoneal macrophages can effectively control abdominal bacterial infection

Abdominal bacterial infection, one of the deadliest causes of sepsis, is polymicrobial and is becoming a common clinical challenge with few treatments [22,111,112]. Shreds of evidence show that mast cells contribute to SIRS by causing hemorrhagic shock, severe tissue damage, polymicrobial sepsis, and trauma-induced bone injury [19,21,112,113]. The local crosstalk between mast cells and the phagocytic effect of PRMs, rather than the local recruitment of neutrophils and monocytes, was revealed in an abdominal polymicrobial septic mouse model that allowed both visualization and conditional ablation of mast cells and basophils in the early stage of abdominal sepsis [112]. During the process of abdominal bacterial infection, the release of pro-inflammatory mediators induced an overwhelming inflammatory response: at first local peritoneal tissue damage and coagulation dysfunction, then acute organ dysfunction, and finally septic shock and death [21,23,111,114]. It indicates that the SIRS caused by abdominal sepsis is closely related to tissue damage, coagulation dysfunction, and SAOD resulting from the actions of the coagulation cascade, fibrinolysis cascade, and cytokines/chemokines, although the specific pathophysiological mechanisms are complex and unclear [19]. In a mouse model of abdominal sepsis after *E. coli* infection, the LPMs not only led to rapid pyrolysis, bacterial clearance, and release of inflammatory mediators, but also polymerized multilayer cellular aggregates with thrombin-dependent fibrin. Therefore, LPM polymerization provided a physical scaffold that effectively controlled the abdominal infection [36]. Compared with monocyte-derived F4/80^−^Tim4^−^SPMs, F4/80^+^Tim4^+^LPMs realized the important functions for the homeostasis of coagulation function [115]. The peritoneal wall of the abdominal sepsis model was analyzed, and it revealed that the F4/80^+^Tim4^+^PRMs were present in the physical scaffold after 4 h of *e. Coli* infection, ranging from small to large (＞8 μm), in dense aggregation [36]. In summary, the role of PRMs in the initiation and development of sepsis is becoming increasingly understood. The mechanisms of PRM activation and regulation may provide new insights into the pathogenesis of sepsis and help researchers identify novel therapeutic targets for this life-threatening condition.

### 1.6 Acute gastrointestinal dysfunction and gut-resident macrophages

#### 1.6.1 Gut-resident macrophages

Gut-resident macrophages (GRMs), characterized by the expression of chemokine receptor CX3CR1, belong to a group of heterogeneous immune cells that reside in the gastrointestinal tract for a long time [116]. Most GRMs are continuously differentiated from Ly6C^high^ monocytes that enter the gastrointestinal via the CCR2-CCL2 axis, regulated by Nr4a1. The remaining GRMs are mainly derived from CX3CR1^high^Ly6C^low^monocytes [79,116]. Ly6C, Ly6G, CD64, CD11b, MHC-II, CX3CR1, and SSC are considered to be the optimal primary markers for detecting the progress of differentiation of monocytes into GRMs in the gastrointestinal tract [35]. GRMs can phagocytose and destroy pathogens and modulate the immune response.

#### 1.6.2 Gut-resident macrophages contribute to the prevention and amelioration of acute gastrointestinal dysfunction

Acute gastrointestinal dysfunction (AGD) induced by sepsis, which promotes the translocation of intestinal bacteria and toxins, contributed to SIRS, the pathological process of sepsis, and SAOD [28,35]. Approximately half of the septic patients had AGD, which was directly related to the increased mortality rate [25,28]. Recent studies have highlighted the importance of GRMs in maintaining gastrointestinal homeostasis, promoting barrier function, and regulating immune responses [35,79,116]. Therefore, it is beneficial for researchers to discover the pathophysiological mechanisms of sepsis-induced AGD based on the research progress of GRMs to further develop prevention and treatment methods. Activation transcription factor (ATF) 4 is a critical transcription factor in the differentiation of bone marrow monocyte/macrophage progenitor cells into bone-resident macrophages (osteoclasts) [117]. In the CLP or LPS-induced septic model, ATF4, a novel regulator of monocyte GRM differentiation, played an important protective role in sepsis-associated AGD. Specifically, ATF4 expression promoted the differentiation of Ly6C^high^ monocytes into GRMs, inhibited the expression of pro-inflammatory cytokines (TNF-α and IL-1β), promoted the expression of CD31 and vascular endothelial cadherin in the gastrointestinal, and inhibited the translocation of intestinal bacteria into lymph nodes and lungs [35].

### 1.7 Sepsis-associated liver dysfunction and kupffer cells

#### 1.7.1 Sepsis-associated liver dysfunction

The liver is a critical organ for immune homeostasis and metabolism, and it is the major injured organ in the early stage of sepsis, especially within 24 h, caused by pro-inflammatory cytokines, sepsis-induced microthrombosis, complement activation, bacterial pathogens, and others, which is commonly known as sepsis-associated liver dysfunction (SALD) and even liver failure (SALF) [13,28,115,118,119]. In epidemiology, sepsis is not considered a major cause of acute liver failure (ALF). However, when it occurs, it is defined as a high-mortality scenario [2]. Both SALD and SALF are mostly characterized by shock with multiple organ dysfunction syndrome (MODS), high bilirubin levels, high proportion of patients with potential liver disease and coagulopathy, high average age cluster, and high proportion of chronic kidney disease [31].

#### 1.7.2 Kupffer cells

The liver is the largest reservoir of macrophages, including KCs, monocyte-derived macrophages, and capsular macrophages, highlighting that the liver is the immunological hub. KCs, the unique liver-resident macrophages, are the major population of liver macrophages (∼90 %), which are derived from yolk sac-derived erythromyeloid progenitors during embryogenesis and undergo self-renewal throughout adulthood [13]. There is increasing evidence that KCs have the function of trapping bacteria and fungi, and that KCs are critical for protecting the liver from injury caused by bacterial transmission, particularly SALD and SALF [13,24,28,[118], [119], [120], [121]].

#### 1.7.3 The role of kupffer cells in sepsis-associated liver dysfunction and liver failure is characterized by a double-edged sword

KCs serve as the gatekeepers for bacteria phagocytosis and endotoxin removal (especially LPS) via STING, interferon regulatory transcription factor 3, TLR4/MyD88/NF-κB, JNK/USP13, and other signaling pathways; at the same time, KCs may be a double-edged sword in that they produce a large number of pro-inflammatory cytokines such as IFN-β, TNF-α, IL-6, and IL-1β), ROS, and lipid superoxide, and recruit other inflammatory cells into the liver, which further initiate local inflammatory cascades, cell death, and oxidative stress that induces irreversible hepatocytes damage and liver injury [24,[121], [122], [123], [124], [125], [126]]. Therefore, to prevent the occurrence of SALD or SALF, the KCs should have effective phagocytosis, phagosome acidification, and bacterial clearance, while producing lower levels of pro-inflammatory cytokines and other mediators that cause liver injury.

Capsules, the outermost antiphagocytic structures of many bacteria and fungi, are critical to evading the recognition and binding by phagocytes and adaptive immunity, which promote intracellular survival, virulence, and translocation of many pathogens [119]. Encapsulated bacteria are the most common septic pathogens in humans and animals, such as *Streptococcus pneumoniae, Klebsiella pneumoniae,* and *Escherichia coli*, which belong to the highly virulent categories of capsular serotypes [119,127]. The liver and its associated KCs were the primary organ and cells targeted by encapsulated bacteria (*Streptococcus pneumoniae* and *Escherichia coli*) in a systematic infection that helped to circumvent the mammalian host's most potent antibacterial machinery against the early stage of sepsis via capsular serotype-specific receptors and complement receptors [119].

Platelets are abundant but short-lived blood cells that have many physiological functions, including hemostasis, vascular integrity, and immunity. Excessive platelet activation caused by sepsis leads to thrombosis and coagulopathy [128]. Most platelet membrane receptors are glycoproteins. The major form of glycosylation is often “capped” by sialic acids, an enzymatic process known as sialylation, particularly α2,3-linked sialic acid [129,130]. Neuraminidase (sialidase), found in many pathogens and mammalian cells, is a glycosidase that catalyzes the hydrolysis of sialic acid junctions and the removal of sialic acids [130]. KCs play an important role in the process of platelet hyperactivation and clearance of desialylated platelets [118,131]. *C*-type lectin domain family 4 member F (CLEC4F), a *C*-type lectin receptor specifically expressed by KCs (CD68^+^KCs), has a high binding affinity for glycoproteins being exposed to β-galactose or β-*N*-acetylgalactosamine [131,132]. A study of platelets based on interstitial and ultrastructural analysis showed that the desialylated platelets induced by bacterial-derived neuraminidases were predominantly phagocytized by KCs in a CLEC4F-dependent manner [131]. This research article provides new insights into the pathogenesis and intervention of sepsis-induced microthrombosis and coagulopathy.

### 1.8 Relative adrenal insufficiency and adrenal gland-resident macrophages

#### 1.8.1 Sepsis and relative adrenal insufficiency

The adrenal gland, composed of a medulla and a cortex, is a key component of the body's stress response system, which can be affected by microbial infections [[133], [134], [135], [136], [137], [138], [139]]. In humans, the cortex can be further divided into three zones: zona glomerulosa, zona fasciculate, and zona reticularis; in mice, the zona reticularis is called the X-zone and exercises sex-dependent regulation, presenting sexually dimorphic organization, size, and functions [140]. Relative adrenal insufficiency (RAI), a risk factor for death and an endotype of sepsis, occurs in 25～60 % of patients with sepsis; and glucocorticoids (GCs) are commonly used in patients with sepsis and septic shock [134,138,139]. However, GC therapy is beneficial to sepsis patients with RAI but detrimental to sepsis patients without RAI. Thus, precision medicine is necessary for the treatment of sepsis: a selective application of GC therapy only for septic patients with RAI [139]. In recent years, by using several advanced imaging modalities, *e.g.*, adrenal heterogeneity in the arterial phase of contrast-enhanced computed tomography (CT), hollow adrenal gland sign on dual-phase contrast-enhanced CT, and adrenal iodine concentration derived from dual-layer spectral detector CT, physicians can predict the outcome of patients during the acute phase of septic shock [133,136,138]. As for the dynamics of sepsis, histological changes in the adrenal glands, including cell destruction and reduction in the absolute area of the zona glomerulosa, the columnar part of the zona fasciculata, and the medulla, can happen in the absence of obvious signs of leukocyte infiltration with venous congestion, which associated with pathophysiological mechanisms such as excessive inflammatory response, hemorrhage, cell death, immune dysfunction, and activation of the hypothalamic-pituitary-adrenocortical axis [134,135,137].

#### 1.8.2 Adrenal gland-resident macrophages

Like other tissue-resident macrophages, adrenal gland-resident macrophages (AGRMs) are derived from the yolk sac during embryonic development, localize in all the regions of the adrenal gland, and play an important role in immune-adrenal interactions [12,63,140]. However, scRNA-seq analysis reveals the heterogeneity of AGRMs, where these cells show sex-restricted distribution and function; and, the maintenance of AGRMs in adulthood is dominated by bone-marrow-derived monocyte recruitment rather than self-renewal; the depletion of AGRMs during stress leads to disturbances to tissue homeostasis, such as dysregulation of local lipid metabolism and reduced local aldosterone secretion [140].

#### 1.8.3 Activation of adrenal gland-resident macrophages worsens the development and progression of relative adrenal insufficiency in sepsis

Sepsis-induced mitochondrial oxidative stress and dysfunction in adrenal glands result in RAI [139]. Nitric oxide (NO) is produced by inducible nitric oxide synthase (iNOS) and causes mitochondrial damage in various tissues; systemic administration of LPS increases iNOS expression and NO production in the adrenal glands; thus, NO contributes to mitochondrial oxidative stress and RAI in sepsis [135,136,141]. Results of double immunofluorescent staining of iNOS with the marker CD31 for adrenal vascular endothelial cells or the AGRM marker CD68 in LPS-induced sepsis demonstrate that iNOS expression is upregulated in adrenal vascular endothelial cells and CD68^＋^AGRMs rather than adrenocortical cells [141]. Accordingly, the overproduction of NO, mainly produced by adrenal vascular endothelial cells and AGRMs, contributes to mitochondrial oxidative stress in adrenocortical cells and subsequently leads to RAI during sepsis. Shreds of evidence indicate that locally induced adrenal inflammation can promote the immune-adrenal crosstalk, which regulates cortisol secretion and even causes RAI, and that immune cells are necessary to mediate the effect of inflammatory stimuli on the steroidogenic pathway [134,137,142]. In a *trans*-well co-culture model of THP1 (human monocyte) derived macrophages and ATC7 zona fasciculata adrenocortical cells, the LPS treatment results in the increased level of *IL-*6 mRNA in ATC7 cells, while the expression levels of key adrenal steroidogenic enzymes, including StAR and DAX-1, are significantly decreased. The treatment with the glucocorticoid dexamethasone prevents the effects of LPS stimulation on the mRNA levels of *IL-6*, *StAR,* and *DAX-1* in ATC7 cells co-cultured with THP-1 cells [142]. Thus, the expression of pro-inflammatory cytokines such as IL-6 and steroidogenic genes in response to LPS is associated with the activation of macrophages via immune-adrenal crosstalk. This theory is further validated by scRNA-seq analysis of the changes in the mouse adrenal transcriptome after fungal sepsis, *i.e.* systemic *C. albicans* infection [143].

### 1.9 Sepsis-associated acute kidney injury and kidney-resident macrophages

#### 1.9.1 Sepsis-associated acute kidney injury

Sepsis-associated acute kidney injury (SA-AKI), preferably defined as the presence of AKI within 7 days of the sepsis onset (diagnosed according to the Global Prognostic Criteria for Improvement in Kidney Disease and Sepsis 3 criteria, respectively), is the most common organ injury (25～75 %) in patients with sepsis and is strongly associated with adverse outcomes, including chronic kidney disease, cardiovascular events, and increased risk of death [2,27,76]. The complex and unclear pathophysiological mechanisms of SA-AKI, mainly including systemic and renal immune inflammation, complement activation, renin-angiotensin-aldosterone system (RAAS) dysregulation, mitochondrial dysfunction, metabolic reprogramming, microcirculatory dysfunction, and macrocirculatory abnormalities, account for the diverse clinical phenotypes and challenging clinical management of SA-AKI [21,27,32,76].

#### 1.9.2 Kidney-resident macrophages

Kidney-resident macrophages (KRMs), which are derived from erythromyeloid precursors during embryogenesis and have the capacity for self-renewal in adult tissues with little or no peripheral blood input, maintain renal homeostasis, monitor the immune microenvironment of the kidney, promote angiogenesis, and control the pathology of AKI and cystic kidney disease [[144], [145], [146]]. KRMs are conserved, complex, and plastic renal-resident mononuclear phagocytes with diverse functions that arise in the yolk sac during early embryogenesis and reside in the kidney during development [144,147]. At the gene transcription level, techniques such as scRNA-seq, have revealed not only the homology between KRMs and other TRMs, such as heart, liver, and lung in terms of origin, life cycle, and even function but also the homology and heterogeneity of KRMs among different species [14,[144], [145], [146], [147], [148], [149]].

#### 1.9.3 Kidney-resident macrophages contribute to the prevention and amelioration of sepsis-associated acute kidney injury

KRMs comprise discrete subpopulations that cannot be conventionally divided. These subpopulations exert varying degrees of influence on anti-inflammatory, pro-angiogenic, and tissue homeostatic in the presence of LPS [150]. Acute systemic *Candida* infections, a common fungal cause of sepsis, are often associated with renal disease, potentially leading to AKI [33,151]. Although CD169^＋＋^KRMs do not directly eradicate *Candida*, they prevent AKI caused by *Candida* infections by promoting the secretion of IFN-γ and the neutrophil ROS response and inhibiting *Candida* growth [151]. *C*-reactive protein (CRP), an acute fluid phase protein, is closely associated with inflammation and infection, particularly sepsis, and can activate macrophages [76]. In the early stage of sepsis, Ly6C^−^KRMs are damaged and can exacerbate SA-AKI in mice; also, intraperitoneal injection of synthetic CRP peptide 1 h after CLP can effectively prevent or control SA-AKI by increasing the number of Ly6C^−^KRMs, enhancing and maintaining Ly6C^−^KRMs ROS production, and changing Ly6C^−^KRMs toward M2-like subtype [33]. F4/80 and CX3CR1 are the primary characteristic markers of KRMs [144,147]. Since F4/80^hi^ KRMs are involved in immunoregulation following injury, the selective depletion of F4/80^hi^ KRMs would exacerbate SA-AKI [32,150]. F4/80^hi^ KRMs express the IL-1 receptor antagonist and restrict the generation of IL-6 in endothelial cells to ameliorate SA-AKI, representing a macrophage-endothelial cell immunoregulatory axis in SA-AKI [32].

### 1.10 Acute pancreatitis and pancreatic-resident macrophages

#### 1.10.1 Acute pancreatitis and sepsis

The pancreas is a long and narrow gland that consists of two main parts: the exocrine gland and the endocrine gland [152]. The exocrine gland is responsible for secreting and excreting pancreatic juice and consists of alveoli and ducts [152,153], while the endocrine gland consists of islets composed of α-cells, β-cells, δ-cells, and *P*P-cells in various sizes, where the primary function is to regulate blood glucose, gastrointestinal motility, pancreatic secretion, and gallbladder contraction [18,152]. Acute pancreatitis (AP), especially severe AP (SAP), characterized by intense local inflammation and SIRS, is an indication for hospitalization in patients with gastrointestinal disease and the leading cause of death in hospitals [26]. Patients with acute necrotizing pancreatitis have an overall mortality rate of 10^～^15 %, and 40^～^70 % of these patients develop a secondary pancreatic infection, sepsis, and even sepsis-associated acute organ injury, with a mortality rate of 80 % [7,26].

#### 1.10.2 Pancreatic-resident macrophages

Pancreatic-resident macrophages (PARMs) are in a heterogeneous family of cells with different origins and phenotypes, characterized by a long half-life and self-maintenance in the adult pancreas [18]. PARMs include exocrine and endocrine resident macrophages that differ not only in phenotype but also in ontogeny and function [152]. In general, the PARM population is mainly derived from yolk sac-derived resident macrophages during embryonic development and fetal liver-derived resident macrophages [10,18,63,109,152,154]. However, studies on PARMs in sepsis and inflammation are extremely limited because the amount of PARMs is very small, and adult pancreatic macrophages are mainly derived from bone marrow-derived infiltrating macrophages or even other TRMs such as ATRMs in healthy states or in pathological states such as AP [18,26,153,155,156].

#### 1.10.3 Activation of pancreatic-resident macrophages worsens the development and progression of acute pancreatitis

The phenotype of PARMs depends on their location, *e.g.* the F4/80^low^ CD11c^＋^subset is found in the islets, whereas the F4/80^hi^ CD11c^－^subset is found in the peripheral islet area [152]. In NF-κB GFP reporter transgenic mice, the activation of the NF-κB signaling pathway in F4/80^hi^ PARMs is induced by the administration of LPS and caerulein-induced AP, and this effect is also associated with NLRP3 inflammasome-induced inflammation in response to pancreatic injury [153]. Islet-resident macrophages (IRMs), a subpopulation of PARMs that senses microbial products in the blood, are in an inflammatory state and express an activation signature characterized by the expression of MHC-II, TNF, and IL-1β at high transcriptional and protein levels [42]. Obesity is a high-risk factor for AP [20,154,155,157]. Although obesity is independent of the recruitment of circulating bone marrow-derived monocytes, it induces local expansion of IRMs. In terms of function, IRMs impair β-cell function in a cell-cell contact-dependent manner [158]. Therefore, it may be helpful for researchers to further investigate the underlying mechanism to explain the role of PARMs in AP and SAP in obese individuals.

### 1.11 Sepsis and splenic-resident macrophages

#### 1.11.1 Spleen and sepsis

The spleen is a complex organ with highly organized compartmentalization and an intricate microcirculatory system. It is a gatekeeper of systemic immunity, initiating and maintaining immune responses to blood-borne pathogens to prevent potentially life-threatening sepsis [127,159]. Splenectomy can lead to serious postoperative complications, particularly sepsis. One possible solution to this medical problem is heterotopic spleen auto transplantation, which restores characteristic splenic architecture and fully reconstitutes monocyte-macrophage, megakaryocyte, and B-lymphocyte populations within 30 days after transplantation [160]. In addition, the spleen is the reservoir for bacteremia in severe community-acquired pneumococcal pneumonia, which is associated with the development of bacteremia and is often associated with sepsis [161]. In sepsis, the spleen has even been implicated in systemic infection, hyperinflammation, and immune dysfunction such as shortened neutrophil lifespan via microbe trapping and spleen-liver axis [162,163]. Thus, the advantages and disadvantages of the spleen in sepsis remain complex and unclear, despite its central role in the blood defense system.

#### 1.11.2 Splenic-resident macrophages

As a result of adaptation to tissue-specific environments, splenic-resident macrophages (SRMs), including red and white pulp macrophages, marginal zone macrophages (MZMs), and marginal zone metallophilic macrophages (MMMs), identified by immunohistochemistry based on localization, morphology, and membrane antigen expression, are extremely heterogeneous [16,[160], [161], [162]].

#### 1.11.3 Splenic-resident macrophages contribute to the prevention and amelioration of sepsis

The splenic marginal zone, including MZMs and MMMs, is a critical microanatomical region for pathogen defense that transmits information about pathogens from the innate to the adaptive immune system [16,162,164]. CD169^＋^SRMs are a subset population of SRMs located in the splenic marginal zone and at the front line of host defense against bloodborne pathogens [16,159,162,165]. Since porcine immunology and the splenic architecture are very similar to those of humans, researchers have conducted a study of early events in the spleen before the onset of bacterial sepsis. Following the injection of *pneumococcus* into an *ex vivo* porcine splenic perfusion model, researchers observed complete clearance of bacteria in the blood and an increase in bacterial counts in the spleen. In a time course study, they further confirmed that each focus of infection originated from the replication of single pneumococcal cells within peri-follicular CD169^＋^SRMs [166]. Similar to the human spleen perfusion *ex vivo* model that was used to study bacterial replication susceptibility, a non-human primate model such as baboon and papio cynocephalus can be used to study splenic involvement during pneumonia, and the mouse model can be used to study experimental intravenous or intranasal *pneumococcal* infection. For instance, in the mouse model, researchers have observed an initial reduction of bacteria in the blood and life-threatening sepsis hours later, representing a population bottleneck driven by efficient clearance of pneumococci by CD169^＋^SRMs, but occasionally accompanied by intracellular replication of bacteria engulfed by CD169^＋^SRMs, where the proliferation of these sequestered bacteria provides a reservoir for dissemination of *pneumococci* into the bloodstream [161,165,167]. Extracellular histones are cytotoxic and have a pro-inflammatory effect [21,93,127]. In systemic candidiasis, microbes are engulfed by the phagocyte receptor SIGN-related 1, which neutralizes myeloperoxidase, facilitating marginal zone infiltration and T-cell-dependent extracellular histone release. Extracellular histones and hyphae induce cytokines in adjacent CD169 SRMs, which selectively deplete mature Ly6G^high^ shortening their lifespan in favor of immature Ly6G^low^ neutrophils that have a defective oxidative burst [162]. Thrombocytopenia is common in patients with sepsis and septic shock, and it is usually associated with adverse clinical outcomes [21,114,131]. The spleen is an important blood filter and stores up to one-third of circulating platelets. And, there is considerable evidence showing that the spleen is involved in sepsis-associated thrombocytopenia [2,16,159,163]. In a study, researchers used confocal microscopy to examine endogenous rat platelets injected intraperitoneally with LPS, and they observed large platelet aggregates in the spleen, where the majority of them were located in the marginal zone and closely associated with CD169^＋^SRMs; the platelet aggregates could interact with infused platelets and contribute to platelet infusion refractoriness. Also, they found that macrophages played an important role in mediating sepsis-associated thrombocytopenia [164]. Moreover, in sepsis or splenectomy, recovery of SRMs function is slow and leads to septic arthritis, SA-AKI, and SALD through the crosstalk mechanism in resident macrophages [144,163,168].

### 1.12 Sepsis and adipose tissue-resident macrophages

#### 1.12.1 Adipose tissue and sepsis

The function of the adipose tissue (AT) is complex, and the disruption of the physiological processes of this tissue can lead to pathophysiological conditions, including infectious diseases and non-infectious chronic diseases, especially in obese populations [82,169]. More and more evidence supports the hypothesis that obesity has an influence on the outcome of septic patients, although the results of clinical studies are controversial and the outcome may depend on the development of obesity-associated metabolic diseases such as diabetes, hyperlipidemia, the severity of these comorbidities, and even the infectious lesions caused by sepsis [5,20,157]. The interaction of the immune system and obesity is critical to the pathophysiology studies of sepsis and other obesity-related comorbidities, although the mechanism by which obesity alters the immune response to pathogens and the mechanism by which infection induces metabolic changes in obese patients are poorly understood [20]. In obese patients with sepsis, *TNF-α mRNA* levels, plasma thiobarbituric acid reactive substances, and protein carbonates are increased in visceral AT, while total plasma antioxidant capacity is decreased [152,154].

#### 1.12.2 Adipose tissue-resident macrophages

AT-resident macrophages (ATRMs) have long been thought to differentiate from local adipose tissue stromal vascular cells and mesenchymal stem cells or from peripheral monocytes that enter the AT by diapedesis, which are thought to be self-renewing and independent of bone marrow progenitors, a well-characterized regulator of AT inflammation [169,170]. Also, ATRMs can support the function of AT and serve as a key inflammatory node for the coordination of immune responses between innate and adaptive immune cells [169]. Therefore, they can induce AT inflammation and insulin resistance, which lead to a corresponding increase in the incidence of obesity and associated comorbidities [169,170]. However, there is also evidence showing that, in amphibians, self-renewing ATMs populate the AT before the establishment of bone marrow hematopoiesis, whereas, in mice, large numbers of ATMs develop from the yolk sac, the mammalian equivalent of the anterior ventral blood island [170,171].

#### 1.12.3 Obesity induces plasticity of adipose tissue-resident macrophage function against sepsis

Sepsis is associated with an increased number of total ATRMs and their M2-like subtype in visceral adipose tissue [152,154]. In a study of obese rats, researchers isolated CD45^＋^/CD68^＋^ATRMs from AT and subsequently cultured them under LPS-mimicking sepsis conditions. They found that CD45^＋^/CD68^＋^ATRMs were abundant in the cultures and they were the major producers of TNF-α. In contrast, the TNF-α/IL-10 ratio was lowest in CD45^＋^/CD68^＋^ATRMs, and it was elevated in both AMs and PRMs, associated with differential activation of the NF-κB signaling pathway. At the same time, obesity makes CD45^＋^/CD68^＋^ATRMs switch to a more pro-inflammatory program [172]. Thus, obesity causes ATRMs to have a plasticity in the responsiveness to sepsis. In addition, high-fat diet (HFD)-induced obesity increases the number of ATRMs and alleviates their apoptosis. However, in an HFD model of severe acute pancreatitis (SAP) induced by intraperitoneal injection of cerulein and LPS, the total number of ATRMs in epididymal AT is reduced, and the polarization of ATRMs leads to the increased proportion of the M1-like subtype [155]. Furthermore, excessive inflammatory responses cause adipocyte lipolysis that produces free fatty acids, which exacerbates ATRM inflammation and the outcome of obesity-associated SAP via the NLRP3-caspase1 inflammasome pathway [156]. This suggests that obesity is an important risk factor for SAP and increases the severity of SAP.

TLRs play a critical role in the immune-inflammatory response associated with sepsis [21,101,125]. For instance, TLR4 is required for HFD-induced production of CD11c^＋^ATRMs and related MyD88-and TRIF-mediated downstream pathways in progenitor cells and AT, which lead to the polarization of CD11c^＋^ATRMs [173]. The expression of TLR4 in adipocytes and ATRMs of AT allows dual activation by lipopolysaccharide and fatty acids and represents a molecular gate between innate immunity and metabolism, which creates local crosstalk between these two cell types and leads to an inflammatory transformation of AT, insulin resistance, *etc* [15,169,170]. The cytokine MIF (also termed MIF-1) and its structural homolog MIF-2 are members of the MIF protein superfamily [17]. Both MIF and MIF-2 are abundantly expressed in adipose tissue and negatively associated with obesity; they may improve insulin resistance and promote wound repair; also, they are highly expressed in systemic inflammation and involved in the pathogenesis of sepsis [17,21,93,108,127]. In LPS mice or septic patients, both MIF and MIF-2 levels were elevated in peritoneal fluid. In contrast, in visceral WAT, MIF expression was increased while MIF-2 expression was decreased. *Mif* gene deletion shifted ATRMs toward the M2-like subtype, while *Mif-2* knockout shifted ATRMs toward the M1-like subtype, and *Mif* deficiency increased the viability of fibroblasts [17]. Therefore, there is an inverse relationship between adipocyte MIF and MIF-2 expression in sepsis, where the downregulation of MIF-2 in AT may increase the polarization of ATRMs toward the M1-like subtype and further drive adipose inflammation.

## 2 Conclusions and perspectives

Sepsis is a rapidly progressive disease and is highly susceptible to the complications of septic shock and SAOD. Although current guidelines for managing sepsis and associated complications are continually being updated and reduce sepsis mortality, septic shock, SAOD, sepsis recurrence, and the presence of comorbidities are independently associated with increased sepsis mortality, particularly in the presence of acute injury to vital organs such as the cardiovascular, brain, kidney, liver, and lung. In addition to systemic life-threatening pathophysiological mechanisms, local mechanisms of organ injury are involved in the development and progression of SAOD. A growing body of evidence confirms that TRMs are involved in sepsis and SAOD. Although the function of TRMs has two sides – angelic (such as CRMs, PVMs, LPMs, GRMs, KRMs, *etc.*) and demonic (such as microglia, SPMs, AGRMs, *etc.*)- due to their phenotype, functional molecules secreted, developmental stage, level of activation, immune microenvironment of the organ in which they are located, and the different stages of the sepsis course. TRMs should be the focus of research for protection against sepsis and SAOD. We should use techniques such as scRNA-seq, *in vivo* cell tracking, and mass spectrometry flow cytometry analysis to deeply characterize TRMs in different organs during sepsis, explore their commonalities such as characteristic markers, and further use protocols such as nanoparticles, *in vitro* cellular interference followed by transfusion, and characteristic inhibitors or agonists to explore TRMs for their potential therapeutic value.

## Funding statement

This work was supported by the 10.13039/501100001809National Natural Science Foundation of China, China (Grant numbers 81871593 and 81701931), and the National Natural Science Foundation of Tianjin, China (Grant numbers 18JCQNJC10500).

## Data availability statement

There is no additional data available for this study.

## CRediT authorship contribution statement

**Yulei Gao:** Conceptualization, Funding acquisition, Supervision, Writing – original draft, Writing – review & editing. **Xin Tian:** Writing – original draft, Writing – review & editing. **Xiang Zhang:** Writing – original draft. **Grace Divine Milebe Nkoua:** Writing – review & editing. **Fang Chen:** Writing – original draft. **Yancun Liu:** Writing – review & editing. **Yanfen Chai:** Conceptualization, Funding acquisition, Supervision, Writing – review & editing.

## Declaration of competing interest

The authors declare that they have no known competing financial interests or personal relationships that could have appeared to influence the work reported in this paper.
